# On the Impact of Artifacts Induced by Mismatches Between Auto‐Calibration Signal and Accelerated 3D GRE Data at 11.7T

**DOI:** 10.1002/mrm.70127

**Published:** 2025-10-18

**Authors:** Joseph Obriot, Franck Mauconduit, Vincent Gras, Chaithya Giliyar Radhakrishna, Maxime Bertrait, Philipp Ehses, Rüdiger Stirnberg, Caroline Le Ster, Nicolas Boulant

**Affiliations:** ^1^ Université Paris‐Saclay, CEA, CNRS, BAOBAB, Neurospin Gif‐sur‐Yvette France; ^2^ MIND Inria Palaiseau France; ^3^ German Center for Neurodegenerative Diseases (DZNE) Bonn Germany

**Keywords:** artifacts, auto‐calibration signal, ultra‐high field

## Abstract

**Purpose:**

The study aims at investigating $B_{0}$ field inhomogeneity artifacts arising from remote locations in the FOV and encountered in accelerated 3D gradient‐recalled echo (GRE) sequences at ultra‐high field, and at providing mitigation strategies.

**Methods:**

Measurements were conducted at 11.7T using a head‐shaped phantom and an accelerated 3D GRE sequence with either integrated or external auto‐calibration signal (ACS) lines. Simulations were performed to reproduce the artifacts. The effects of varying GRAPPA reconstruction parameters (kernel size and regularization) were also examined.

**Results:**

$B_{0}$ field inhomogeneities located outside the $B_{0}$ shimmed region of interest (i.e., the brain) were observed to return ripple‐like artifacts within this region, particularly at long echo times. The simulation results support these findings, and the idea that the observed artifact originates from a mismatch between ACS and accelerated data due to intra‐voxel dephasing at different resolutions (ACS lines having an intrinsically lower resolution). The short echo time enabled by external (i.e., preacquired) ACS lines reduced artifacts compared to integrated ones. Varying GRAPPA kernel sizes and increasing the number of ACS lines can improve image quality, yet without full compensation.

**Conclusion:**

This study highlights ripple‐like artifacts amplified with field strength and arising from a lack of coherence between the ACS and imaging (3D GRE) signal caused by $B_{0}$ intra‐voxel dephasing. To minimize these artifacts, care should be taken in order to preserve the relevant information in the ACS data to properly compute the GRAPPA kernels.

## 1 Introduction

The ubiquitous use of MRI in neuroscience has driven the pursuit of increasingly higher main magnetic fields ($B_{0}$) to improve the image spatiotemporal resolution by trading off signal‐to‐noise ratio (SNR) gain. Ultra‐high fields of 7T are now common in research, while unique MRI scanners going as high as 10.5T and 11.7T are operational and have already shown success in producing images in vivo [1, 2] and in vitro [3].

As the main field increases, however, challenges emerge, in particular the pronounced inhomogeneity of the $B_{1}^{+}$ and $B_{0}$ fields. Specific absorption rate (SAR) is also a major concern for most sequences.

Much progress has been made to mitigate artifacts induced by $B_{1}^{+}$ field inhomogeneity, such as with parallel transmission methods—calibration‐free or subject‐tailored—yielding homogeneous excitation profiles [4]. $B_{0}$ field offsets ($\Delta B_{0}$, mostly originating from imperfections in the main magnetic field and susceptibility differences between air and tissue) are mitigated by shimming within a volume of interest (usually set to the brain in neuroimaging). Inside the brain, the standard deviation of $\Delta B_{0}$ is around 80 Hz at 11.7T [1], but notable large excursions occur close to air cavities, reaching several hundreds of Hz. Functional MR images, that typically require $T_{2}^{*}$‐weighted gradient‐recalled echo‐planar imaging (EPI), are thus notoriously difficult to acquire in brain regions nearby sinuses and ear canals due to the loss of signal induced by $B_{0}$ gradients ($\nabla B_{0}$), that is, intra‐voxel dephasing. But also, structural $T_{2}^{*}$‐weighted imaging or $T_{2}^{*}$ mapping, susceptibility‐weighted imaging, or quantitative susceptibility mapping using gradient‐recalled echo (GRE) acquisitions, can suffer from such signal loss.

In particular, high‐resolution structural and quantitative GRE imaging is preferably performed using 3D sequences, where phase encoding is performed along two directions. This is primarily to maximize SNR [5, 6, 7, 8], but further reasons include facilitating very high slice resolution and efficient use of parallel imaging [9] along two phase encode (PE) directions. While reducing voxel size is a very effective means to counteract intra‐voxel dephasing, even high‐resolution GRE imaging can suffer from $\nabla B_{0}$‐induced artifacts [1].

There is similarly a desire to reduce the acquisition time (TA) to mitigate artifacts induced by variations during the acquisition, such as the subject's motion. A common way to achieve this is to employ parallel imaging with an acceleration technique such as GRAPPA [10]. The latter consists of skipping k‐space lines along the PE direction(s), thereby leading to a violation of the Nyquist criterion and yielding an aliased image. In addition, a Nyquist‐sampled k‐space region, known as the auto‐calibration signal (ACS), is required. To this end, additional lines in the center of k‐space are often acquired. ACS data can be internal—coming from the same acquisition as the aliased image data—or external, that is, acquired in a pre‐scan with minimized echo time (TE) and repetition time (TR) and therefore usually in a short amount of time to minimize scan time and motion artifacts. Since the ACS typically covers only a small portion of k‐space compared to the full resolution acquisition, it inherently has a much larger voxel size, making it more susceptible to signal loss from intra‐voxel dephasing, which can propagate across the image due to acceleration.

**FIGURE 1:**
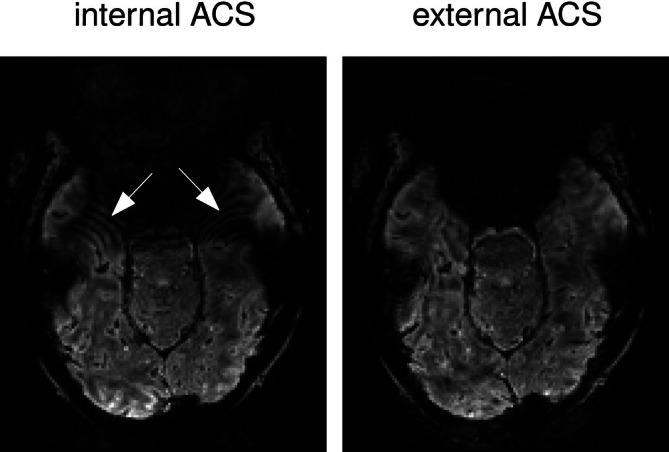
Illustration of the ripple artifact encountered during initial tests in 3D GRE scans acquired with iPAT = 2×2.

On the path to functional MRI at 11.7T, tests were initially performed for quality control at the target resolution of 1.2 mm. These tests showed non‐trivial artifacts, which led us to simplify the acquisition with a 3D GRE sequence in order to troubleshoot the problem. Undersampled 3D GRE images thereby revealed likewise ripple artifacts (Figure 1) which in turn led to this investigation and study.

The aim of this study is thus to investigate artifacts introduced by intra‐voxel dephasing, which causes a mismatch between low‐resolution ACS and high‐resolution undersampled data in accelerated 3D GRE. Simulations were performed to support the theory. We finally provide another scenario in which mismatches between ACS and accelerated data arise from the use of different RF pulses in the two acquisitions, similarly resulting in artifacts.

## 2 Methods

### 2.1 Observations

Measurements were performed on the Iseult 11.7T scanner with an 8Tx‐31Rx RF coil [11, 12] driven in a circularly polarized (CP), slab‐selective mode and on a realistic head‐shaped phantom [13]. An axial 3D GRE sequence was used with TR = 40 ms, TE = 4 or 20 ms, resolution = 1.2×1.2×1.2 mm^3^, matrix size = 144×120×160, flip angle $=10^{\circ }$, left‐right phase encoding. This sequence was acquired 3 times: with and without acceleration (2×2 GRAPPA, respective TA = 3 min 07 s and 11 min 31 s), integrated or external ACS (24×24 lines).

The phantom is based on [14] with a two‐compartment design made to attempt reproducing the human head and neck geometry.

### 2.2 Simulations

**FIGURE 2:**
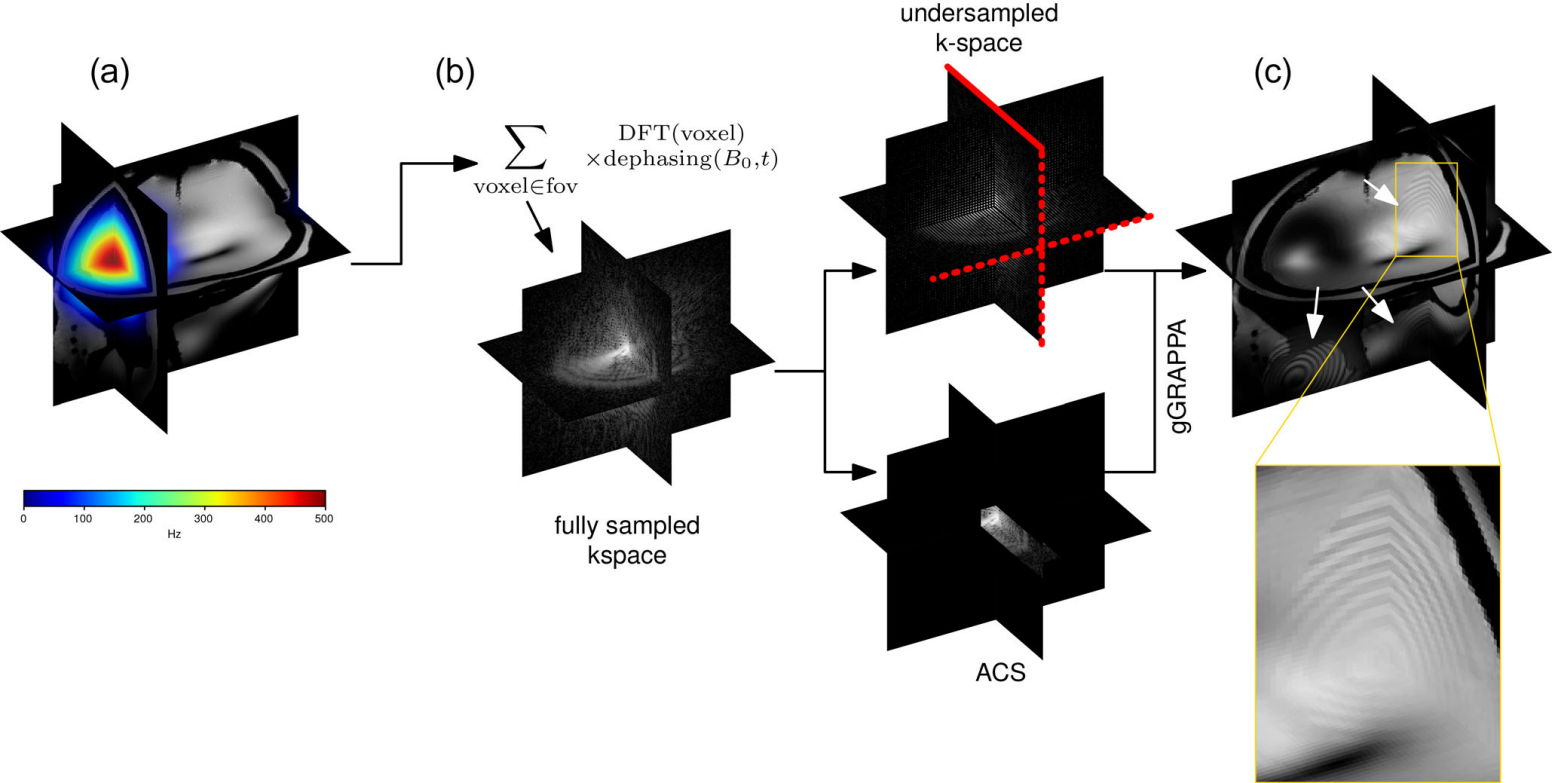
Artificial induction of ripple artifacts due to $B_{0}$ field inhomogeneity in accelerated 3D GRE. Base image is acquired with TE = 4 ms, Equation (3) is applied with a simulated TE of 20 ms using a synthetic $B_{0}$ map overlayed on the magnitude, fully sampled image, shown in A. The reconstruction process is shown in B and the resulting image in C, acceleration directions are shown with dotted red lines (2×2), and the read direction with a solid red line. Ripple artifacts resulting from the remote $B_{0}$ gradient area are visible at the three aliased corners, away from the strong $B_{0}$ offsets.

Simulations were conducted to artificially reproduce the ripple artifacts by applying both inter and intra‐voxel dephasing to a fully sampled, artifact‐free acquisition performed with a TE of 4 ms. A synthetic $\Delta B_{0}$ field was applied with a peak set to 500 Hz above the sinuses, decreasing spatially in a Gaussian manner as shown in Figure 2A, to transform the TE = 4 ms data in order to mimic dephasing occurring at TE = 20 ms.

The inter‐ and intra‐voxel dephasing along the readout direction after partition or phase encoding at a voxel level was calculated using a first‐order decomposition of the $B_{0}$ field:

(1) $$S(t,k_{y},k_{z})={∭}_{r_{0}-\Delta r/2}^{r_{0}+\Delta r/2}\rho \left(r\right)e^{i(k\cdot r)}e^{i\left(\Delta B_{0}+\nabla B_{0}\cdot (r-r_{0})\right)t}\;dr$$

where t is the time after the excitation pulse, r is the position vector (x,y,z) centered inside a voxel at position $r_{0}$ and size Δr, with its associated $\Delta B_{0}$ and $\nabla B_{0}$. The encoding steps k are defined as $\left(k_{x}(t),k_{y},k_{z}\right)$ with $k_{y}$ and $k_{z}$ corresponding to the two phase‐encoded spatial frequencies, and the readout along x given by:

(2) $$k_{x}(t)=-\gamma \int_{0}^{t}G_{x}(u)\;du$$

After integration [15], the signal can be further expressed in terms of the ideal (without $\Delta B_{0}$ offset) signal $S_{0}$ for each voxel as

(3) $$\begin{array}{ll} S(t) & =S_{0}(t)\times e^{i\Delta B_{0}t}\\ & \;\times \underset{d\in [x,y,z]}{\prod }\frac{\text{sinc}\left((k_{d}+{\left[\nabla B_{0}\right]}_{d}t){\Delta r}_{d}/2\right)}{\text{sinc}(k_{d}{\Delta r}_{d}/2)} \end{array}$$

The ratio of sincs above arises from the fact that we isolate the $\nabla B_{0}$ impact as a multiplicative factor to the undisturbed signal (when $\nabla B_{0}=\Delta B_{0}=0$, $S(t)=S_{0}(t)$). A consequence of that expression is that the fully sampled magnitude image experiences not only a signal attenuation at $k_{x}=0$, but also certain high‐frequency components along the readout direction can be distorted due to the shape of the sinc function, thus potentially leading to ripples in the same direction.

In the simulation, Equation (3) was applied to the TE = 4 ms k‐space data, 24×24 central lines were extracted to serve as the integrated ACS, while a 2×2 accelerated dataset was simulated by undersampling the full k‐space 2×2‐fold. Offline image reconstruction was performed using gGRAPPA [16] and images of the individual channels were combined using root‐sum‐of‐squares.

### 2.3 Manipulation of ACS Lines

To confirm the effects of intra‐voxel dephasing causing mismatches between low‐resolution ACS data and the higher‐resolution accelerated acquisition, we reconstructed images using undersampled data acquired at TE = 20 ms, and ACS lines extracted from fully sampled TE = 4 ms image (24×24 lines). In addition, in the ACS image, a portion of the FOV (phantom neck) was artificially reduced in magnitude at varying levels.

The associated reconstructions were compared to the fully sampled image acquired at TE = 20 ms using the structural similarity index measure [17] (SSIM).

### 2.4 GRAPPA Reconstruction Parameters

The accelerated image acquired at TE = 20 ms with internal ACS was reconstructed offline with gGRAPPA using different GRAPPA kernel sizes and regularization parameter (λ) to assess the possibility within this reconstruction framework to compensate for the artifacts. Reconstruction was also performed using varying numbers of integrated ACS lines. Default parameters were set to: kernel size = (4, 4, 5) (partition, PE, readout), $\lambda =10^{-4}$, number of ACS lines = 24×24. The magnitude images were again compared to the fully sampled image using the SSIM.

### 2.5 Mismatch Caused by Difference in RF Pulse Excitation

RF field maps were measured using a magnetization‐prepared turbo flash sequence at 11.7T with 5 mm isotropic resolution and TR = 15 s [18]. A nonselective parallel transmission (pTx) pulse (target flip angle = 10°) was calculated from the masked phantom brain using the fast gradient ascent pulse engineering (fastGRAPE) method [19, 20], which yielded a much improved flip angle homogeneity versus CP (normalized root mean square error, NRMSE = 4.5% versus 40.7%). The RF pulse was then inserted in the 3D GRE sequence to acquire fully sampled images at TE = 3 and 20 ms (TA = 15 min 22 s). The acquisition at TE = 3 ms was repeated in the CP mode of excitation. In simulation, reconstruction of the undersampled (iPAT = 2×2), TE = 20 ms pTx data was performed separately with pTx ACS and CP ACS data extracted from the TE = 3 ms acquisitions, thereby exploring the impact of using distinct RF pulses in the two sub‐acquisitions.

## 3 Results

**FIGURE 3:**
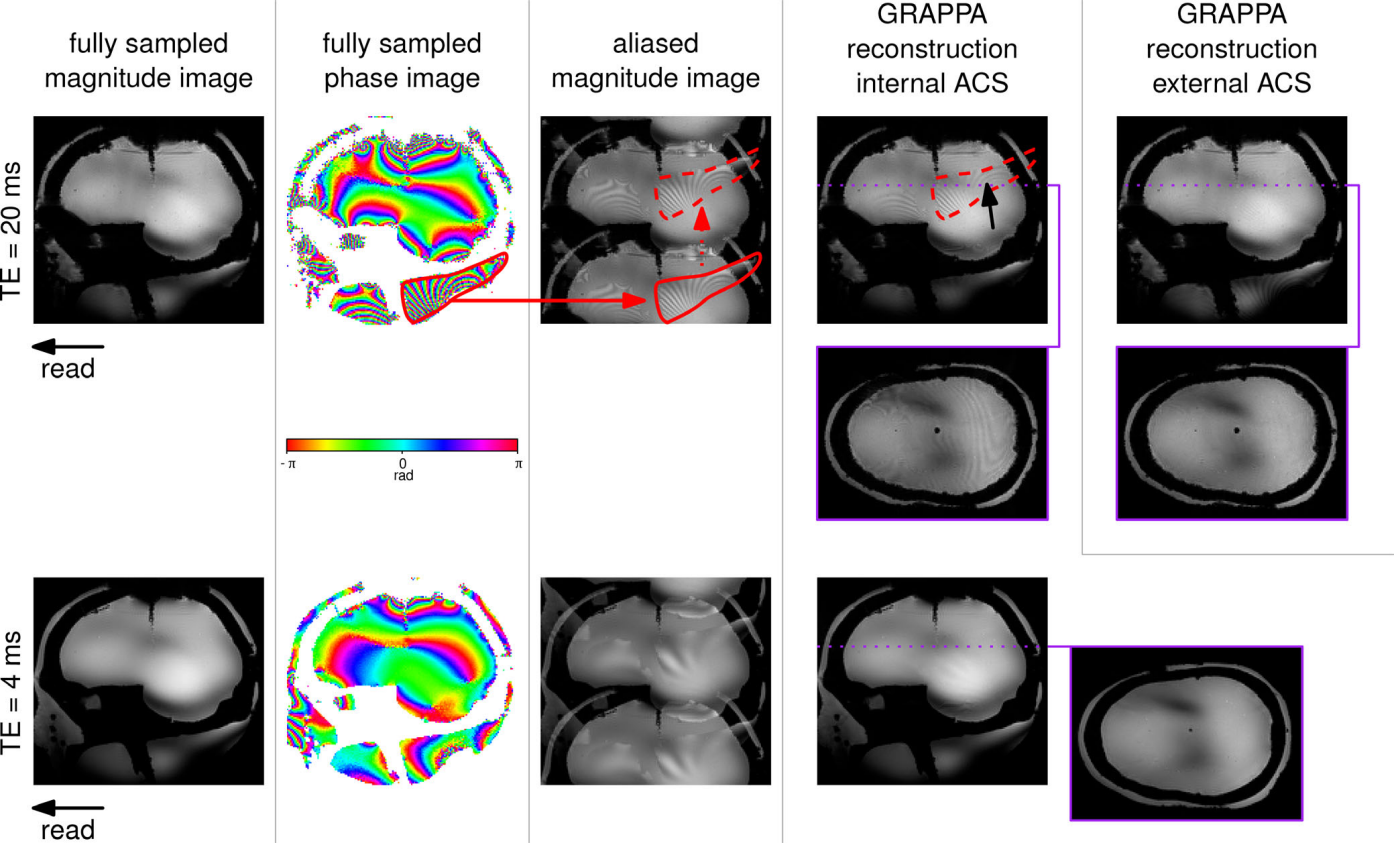
Aliasing effects of $B_{0}$ field inhomogeneities on GRAPPA reconstructions: examples of rapid dephasing areas and their aliased phantom in accelerated data are delineated with solid and dashed red lines, respectively. Ripple artifacts originating from a remote $B_{0}$ field gradient are still visible after GRAPPA unfolding at TE = 20 ms and to a lesser extent at TE = 4 ms, with a decreased spatial frequency. Switching to external ACS for GRAPPA calibration significantly reduces artifacts in the long TE acquisition.

Non‐accelerated and accelerated (iPAT 2×2, 24×24 ACS lines) magnitude and phase images acquired at TE = 4 and 20 ms are shown in Figure 3, along with aliased images. As expected, the non‐accelerated images show few artifacts due to $\nabla B_{0}$ beside signal loss (worse at TE = 20 ms) at the interface between air and the top of the phantom “brain”, as well as in the “neck”. Increased dephasing at longer echo time can also be visualized on the phase images (the $\exp(i\Delta B_{0}t)$ term in Equation (3)), and the repercussion on the aliased image is visible. The complex values with rapidly changing phases fold onto a shimmed area (the “brain”), introducing interference patterns. The GRAPPA unfolding process is able to correct most but not all of the ripples visible on the aliased image, depending on the accuracy of the kernels evaluated with lower resolution ACS data.

The integrated ACS reconstructions at TE = 20 ms show an increase in the frequency of the ripples compared to TE = 4 ms, as suggested by the inter‐voxel dephasing term, and an increase in the residual artifacts after GRAPPA unfolding. Meanwhile, the external ACS reconstructions show no such artifacts. The echo time of external ACS lines is significantly shorter than the TE of the imaging scan, as they are designed not to preserve contrast but to be acquired as quickly as possible with the shortest TE, thereby minimizing intra‐voxel dephasing.

**FIGURE 4:**
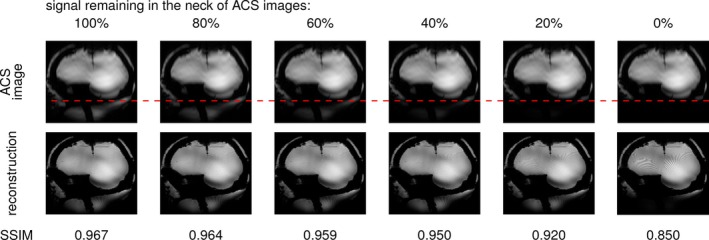
Effects of manually attenuating the neck signal in ACS lines to mimic intra‐voxel dephasing. The first row shows the ACS images used for the reconstruction below at higher resolution. The ACS are extracted from a TE = 4 ms fully sampled acquisition, while the undersampled data are extracted from a TE = 20 ms acquisition (the same as in Figure 3). The dashed red line indicates the slice below which the signal is artificially attenuated.

Figure 4 shows the reconstruction from undersampled data at TE = 20 ms, with ACS lines borrowed from the 24×24 center k‐space from a fully sampled TE = 4 ms acquisition, similar to an external, short‐TE, ACS pre‐scan. The resulting reconstruction in the left‐most column presents minimal artifacts. At this stage, with a TE of 4 ms, the ACS lines still contain a signal in the neck. As we progressively and artificially attenuate the neck signal from the ACS images to mimic intra‐voxel dephasing, we observe the gradual emergence of ripple artifacts in the shimmed brain region originating from a remote area with a high $B_{0}$ gradient.

As seen in Figure 2C, adding $B_{0}$ field inhomogeneities to an initially artifact‐free TE = 4 ms image, then simulating from Equation (3) a 20 ms echo time and finally reconstructing the artificially undersampled image using GRAPPA, yields the same kind of artifacts as in Figures 3 and 4, appearing at the aliased locations of the added $B_{0}$ offsets.

**FIGURE 5:**
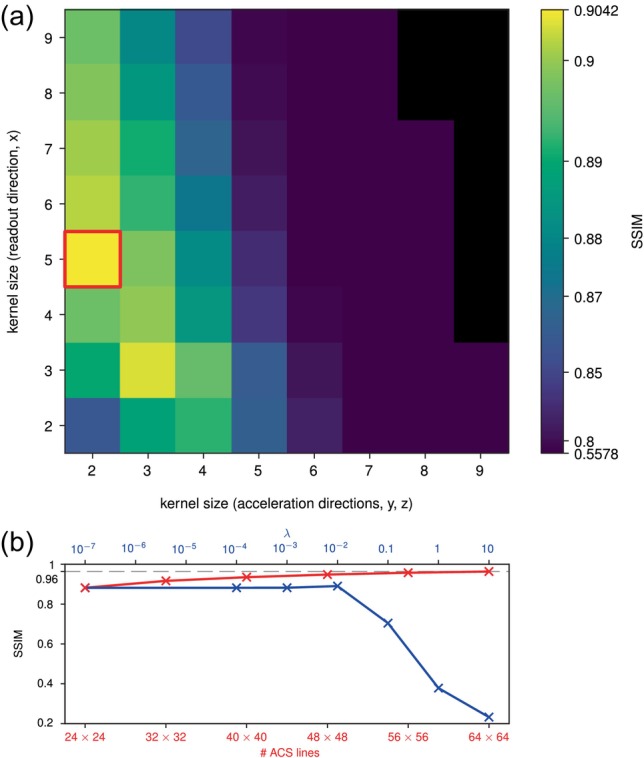
Influence of GRAPPA parameters. The effects of kernel size [A], number of ACS lines, and λ [B] for acceleration factor 4 (2×2) are shown with the SSIM and with reference to the fully sampled image (TE = 20 ms). The red contours indicate the maximum SSIM at kernel size = (2, 2, 5). Kernel sizes for which reconstruction failed are shown in black.

Given the severity of the artifacts being reconstruction‐dependent, we show in Figure 5 how varying GRAPPA reconstruction parameters in a TE = 20 ms acquisition with internal ACS affects image quality by computing the SSIM against the fully sampled image. The kernel size used for the reconstruction was varied in all three dimensions. However, since the results exhibited near diagonal symmetry in the two acceleration directions, for clarity, we present the resulting SSIM along the diagonal, where the maximum values occur. The regularization parameter λ was varied logarithmically from $10^{-7}$ to 10. The image quality change caused by λ was marginal below the threshold λ=0.01, above which the reconstruction quickly fails at unfolding the image, leading to a sharp drop in SSIM. In contrast, increasing the number of internal ACS lines shows a monotonic increase in image quality and reduction in ripples. However, the SSIM values remain lower than those achievable with external ACS (Figure 4) unless the number of internal ACS lines is increased to the point where the benefit of acceleration is effectively lost, thereby compensating for intra‐voxel dephasing with higher ACS resolution.

As the resolution of the ACS tends to reach the resolution of the undersampled data, the SSIM converges to a value slightly lower than 1, explained by the fact that ACS kernels are evaluated in a least squares sense by exploiting the complementarity between profiles across the receive channel, which are thus unable to retrieve the exact original image.

**FIGURE 6:**
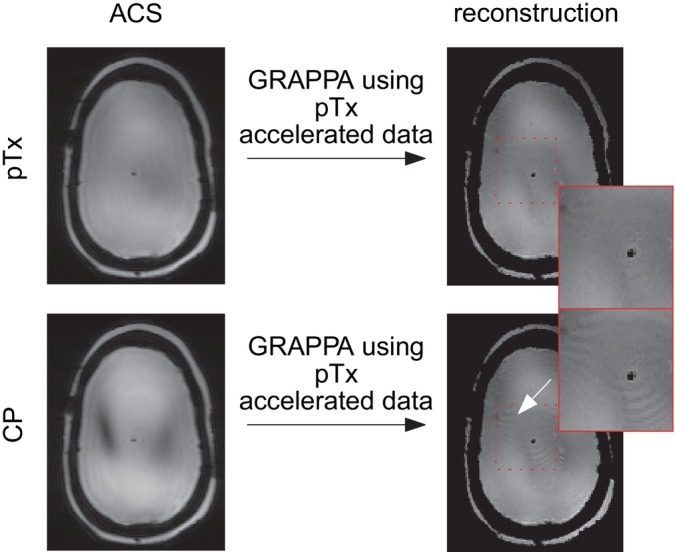
Effect of a mismatch between the ACS and accelerated data caused by different RF excitation pulses. ACS data are composed of 36×36 lines of a TE = 3 ms acquisition, while accelerated pTx data (iPAT 2×2) comes from a TE = 20 ms acquisition.

Figure 6 shows that this artifact can also be caused by mismatches between ACS and accelerated data acquired with different RF pulses. While the $B_{1}^{+}$ field inhomogeneity becomes much more severe at ultra‐high field, especially at 11.7T, finding an RF shim mode that completely avoids regions with extremely weak $B_{1}$ values is extremely challenging if not impossible. If these weak values are present in the ACS data acquisition, this can cause errors in the GRAPPA kernel estimations when the accelerated data acquisition is performed with parallel transmit pulses that mitigate $B_{1}$ field inhomogeneity.

## 4 Discussion

In a typical accelerated acquisition, ACS data have an intrinsically lower resolution than the undersampled data in the PE directions. This makes ACS data more susceptible to intra‐voxel dephasing, inducing a much larger loss of signal in the high $B_{0}$ gradient areas, unless they have a short TE, like with external ACS. Such differences between the ACS lines and the undersampled data lead to errors in the GRAPPA kernels (or receive sensitivity profiles in SENSE [21]), preventing proper unfolding of the aliased voxels due to rapid signal variations. This issue is particularly pronounced at ultra‐high fields.

Since to the signal in Equation (3) is dependent on echo time and $B_{0}$ field inhomogeneities, we expect the same issue to occur at lower fields when the TE is proportionally increased (as shown in Figure S1).

The same figure also demonstrates that increasing the resolution does not mitigate the problem since it widens the “intra‐voxel dephasing” gap between the ACS and the final undersampled data. Importantly, in this case, the effect can also originate from large $B_{0}$ offset areas located outside of the shimmed region of interest. This can include the neck (with usually a poor $B_{0}$ shim due to the presence of the vertebra), the subject's face (particularly the mouth for subjects holding braces). A mismatch between the low‐resolution ACS images and the undersampled images impairs the ability of GRAPPA to properly unfold GRE volumes. As a consequence, even shimmed areas that are aliased with high $B_{0}$ offset regions are not completely immune to the ripples introduced by inter‐voxel dephasing.

The fast acquisition and short‐TE nature of external ACS lines, on the other hand, makes them relatively immune to the loss of signal induced by intra‐voxel dephasing. This signal preservation increases the coherence between the high‐resolution image and the ACS, allowing GRAPPA to better unfold the former compared to using integrated ACS. As demonstrated in Figure 4, signal losses in the ACS are acceptable as long as one is above a certain threshold, justifying the potential contrast differences between ACS and accelerated data. However, as shown in Figure 6, a mismatch between ACS and accelerated data can have other origins, such as the use of different RF pulses, leading again to artifacts. It is currently the case in the default pTx‐enabled 3D GRE sequence on our MRI system: ACS with CP, undersampled data acquisition with pTx.

ACS lines acquired externally usually have a much shorter TR than the subsequent imaging scans, which can be a serious limitation due to SAR restrictions. The pTx pulses are typically designed to work optimally and within SAR limits during the longer imaging TRs. To stay within SAR limits during short ACS TRs, using the same pTx pulses would enforce very low ACS flip angles. RF pulses in CP mode can be more beneficial in the ACS in terms of SAR and shorter TR, but can lead to this kind of mismatch. While it is not clear how dramatic this can be for brain acquisitions at 7T, the severity of the $B_{1}$ field inhomogeneity problem at 11.7T makes it important to consider in future experiments.

If integrated ACS cannot be avoided, these artifacts can be mitigated by, for example, selecting the acceleration directions so that areas with high $B_{0}$ gradients are not aliased into the region of interest. However, this approach is challenging in practice due to the anatomy of the human head and the location of cavities, particularly the paranasal sinuses and ear canals. It also becomes increasingly difficult as the acceleration factor increases. The most effective strategy is to avoid exciting areas with high $B_{0}$ gradients by employing slab selection to focus on the region closest to the shim volume, for example, to eliminate neck‐induced artifacts. As a result, for integrated ACS, we cannot propose a general solution but only mitigation strategies that must be fine‐tuned on a case‐by‐case basis.

GRAPPA reconstruction parameters such as kernel size and regularization parameter can help mitigate these artifacts. In Figure 5, the optimal kernel size was found to be (2, 2, 5), suggesting that a smoother estimation of the receive sensitivity profiles in the undersampled PE directions via a smaller GRAPPA kernel size compared to the readout direction may help filter out fast signal variations. However, the SSIMs obtained with these various parameter combinations could not match those achieved with external ACS. Increasing the number of ACS lines naturally helps as well, as it provides higher‐resolution ACS data and thereby reducing intra‐voxel dephasing and, consequently, the mismatch.

The artifact highlighted in this study has implications beyond 3D GRE acquisitions, which was considered here as a textbook case sequence for simplicity. 2D and 3D EPI sequences for fMRI also typically have long TE to optimize BOLD sensitivity. Even though EPI ACS is typically acquired externally by default, widely used vendor implementations use the same EPI train for ACS as for subsequent accelerated imaging, only with disabled data acquisition of the outer k‐space lines. As this implies the same long echo time (and repetition time) but with larger voxels, such vendor sequences are particularly susceptible to the discussed kind of artifacts. Thus, beyond reducing sensitivity losses due to respiration and motion, our proposed mitigation strategies can also be applied to address these issues in such sequences, but with increased complexity due to the tight coupling of echo time and number of k‐space lines acquired per shot. For instance, while short‐TE 3D GRE ACS can be beneficial for EPI, one may have to additionally consider the geometric mismatch between ACS and accelerated EPI, especially since areas affected by such distortions are also affected by spatially fast varying phases. This is relevant even when choosing EPI‐based ACS. For instance, in low PE acceleration settings (e.g., in a wide range of typical fMRI applications), the ACS may be acquired with a single shot and thus mismatched distortions. In higher PE acceleration settings (e.g., high‐resolution EPI), the ACS and EPI PE bandwidth are typically matched by switching the ACS to interleaved multi‐shot EPI. In this case, the ACS minimum achievable echo time yet automatically increases as the ACS voxel size decreases and the acquisition becomes more sensitive to physiological noise, in particular in 2D‐EPI. This can be mitigated to a high degree by using the FLEET [22] method, which proposes, among other improvements, a minimized TE and TR that makes multi‐shot EPI ACS also less susceptible to intra‐voxel dephasing.

This study was performed on a phantom on purpose to avoid confounds from other phenomena (e.g., motion, breathing) and to more clearly demonstrate the artifacts on simple objects under well‐controlled conditions, to be replicated in vivo (Figure 1).

## 5 Conclusions

We have investigated GRAPPA reconstruction artifacts in $B_{0}$‐shimmed accelerated 3D GRE acquisitions of the brain at ultra‐high field. Inhomogeneities of the main magnetic field cause dephasing, leading to ripples in the aliased regions of the undersampled image. These ripple artifacts should ideally be eliminated during GRAPPA unfolding, but the mismatch between the lower resolution ACS and the accelerated data impairs this process. This mismatch is primarily due to the difference in signal loss from intra‐voxel dephasing, with the ACS losing much of the signal in high $B_{0}$ offset areas. Coping strategies include using an external—short‐TE—ACS scan, slab‐selective excitations to avoid acquiring the MR signal originating from high‐$\nabla B_{0}$ regions, tuning of the reconstruction kernel size, increasing the number of ACS lines, and optimizing the frequency encoding direction.

## Supporting information




**Figure S1**: The same ripple artifacts showing in various acquisition parameters. 11.7T images were acquired with a TE of 20 ms and isotropic resolutions, 7T was acquired with a TE made to match intra‐voxel dephasing of the 11.7T field (20×11.7/7≈34ms). All data were reconstructed with 24×24 ACS lines. At 11.7T the acquisitions were performed with pTx, while the ones at 7T were performed in CP mode.
