# Supplementary material for: On the Impact of Artifacts Induced by Mismatches Between Auto‐Calibration Signal and Accelerated 3D GRE Data at 11.7T
Source: Magn Reson Med. 2025 Oct 18;95(3):1440–7. doi: 10.1002/mrm.70127 (PMC12746375; doi:10.1002/mrm.70127)
Supplement: Supplementary file 1 — Figure S1: The same ripple artifacts showing in various acquisition parameters. 11.7T images were acquired with a TE of 20 ms and isotropic resolutions, 7T was acquired with a TE made to match intra‐voxel dephasing of the 11.7T field (20×11.7/7≈34ms). All data were reconstructed with 24×24 ACS lines. At 11.7T the acquisitions were performed with pTx, while the ones at 7T were performed in CP mode. [file MRM-95-1440-s001.pdf]

Supporting Information: On the impact of artifacts induced by mismatches between auto calibration signal and accelerated 3D GRE data at 11.7T

August 27, 2025

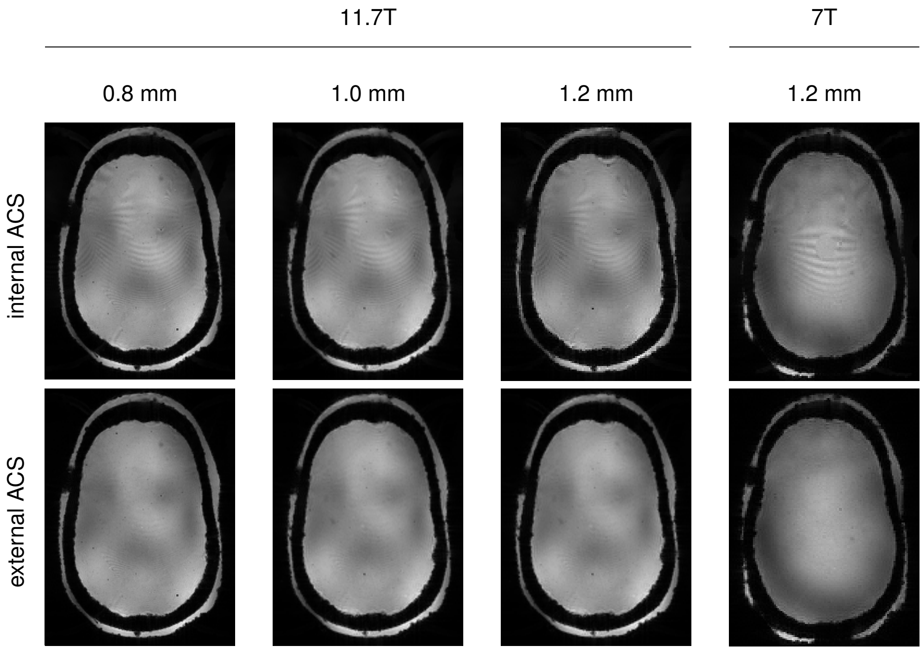

Figure S1: The same ripple artifacts showing in various acquisition parameters. 11.7T images were acquired with a TE of 20 ms and isotropic resolutions, 7T was acquired with a TE made to match intra-voxel dephasing of the 11.7T field ($20 \times 11.7/7 \approx 34$ ms). All data were reconstructed with 24×24 ACS lines. At 11.7T the acquisitions were performed with pTx, while the ones at 7T were performed in CP mode.
